# The effect of immunoregulatory bacteria on the transcriptional activity of Foxp3 and RORyt genes in the gut-associated lymphoid tissue with *Salmonella*-induced inflammation in the presence of vancomycin and *Bacteroides fragilis*

**Published:** 2020-06

**Authors:** Yuliia Bukina, Marina Thyhonovska, Mariya Koval, Mariya Marushchak, Inna Krynytska, Aleksandr Kamyshnyi

**Affiliations:** 1 Department of Microbiology, Virology and Immunology, Zaporizhzhya State Medical University, Zaporizhzhya, Ukraine; 2 Department of Normal Physiology, Zaporizhzhya State Medical University, Zaporizhzhya, Ukraine; 3 Department of General Chemistry, I. Horbachevsky Ternopil National Medical University, Ternopil, Ukraine; 4 Department of Functional and Laboratory Diagnostics, I. Horbachevsky Ternopil National Medical University, Ternopil, Ukraine

**Keywords:** *Salmonella*, Vancomycin, Bacteroids, Immunoregulation, Genes expression

## Abstract

**Background and Objectives:**

Intestinal microbiota is involved in the development and maintenance of immune homeostasis. This study was conducted to investigate the levels of key immunoregulatory bacteria in the intestinal wall-associated microflora and its effect on the transcriptional activity of the Foxp3 and RORyt genes in the gut-associated lymphoid tissue (GALT) of rats with *Salmonella*-induced inflammation, both untreated and treated with vancomycin and *Bacteroides fragilis*.

**Materials and Methods:**

To determine the levels of immunoregulatory bacteria in GALT of rats Q-PCR was used to identify them by species-specific 16S rDNA genes. Transcriptional activity of Foxp3 and RORyt genes was determined using Q-PCR with reverse transcription.

**Results:**

In animals treated with both vancomycin and *Salmonella*, the levels of segmented filamentous bacteria (SFB) increased while *Akkermansia muciniphila* and *Faecalibacterium prausnitzii* decreased. In rats that received pretreatment with vancomycin and then were infected with *S.* Enteritidis and *S.* Typhimurium, the levels of *SFB* increased, and the number of *Bacteroides-Prevotela* group, *A. muciniphila, Clostridium* spp. clusters XIV, IV, and *F. prausnitzii* significantly decreased, decreasing Foxp3 and increasing Rorγt mRNA expression. Administration of *B. fragilis* to animals treated with *S.* Enteritidis or *S.* Typhimurium and pre-treated with vancomycin caused a decrease in *SFB* and Rorγt mRNA levels and conversely, increased the numbers of the *Bacteroides-Prevotela* group, *Clostridium* spp. clusters XIV, IV, *A. muciniphila, F. prausnitzii* and Foxp3 gene expression in GALT.

**Conclusion:**

Our results suggest that the commensal microorganism *B. fragilis* may provide a protective role against the development of experimental colitis, which has to be taken into consideration for further clarification of the effective therapeutic strategy of inflammatory bowel diseases, irritable bowel syndrome and necrotising colitis.

## INTRODUCTION

Human intestinal microbiota and intestinal physiology are results of millions years of co-evolution and adaptation. Dysbiosis of microbiota is now being increasingly recognised as a salient feature, cause, or effect in immune disorders. Epithelial cells express a range of immune pattern recognition receptors (PRR), including various Toll-like receptors (TLR) and nucleotide-binding oligomerization domain-like receptors (NLR). Triggering of PRR signalling by pathogenic bacteria, or commensal species under inflammatory conditions, promotes a range of immune responses, which limit further invasion and clear infected cells. It can also promote the local release of pro-inflammatory cytokines, which can disrupt epithelial tight junctions leading to a ‘leaky’ barrier and allowing the ingress of opportunistic bacterial species (1–6).

Upon colonisation of the intestine, bacteria and bacterial products are recognised by receptors, including TLR and NLR, not only of the epithelial, but also of the mucosal immune system cells which direct appropriate pro-inflammatory or tolerogenic immune responses (5–7). CD4 T cells coordinate diverse immune responses to deal with various pathogens. Binding to the T cell receptor (TCR) activates naive CD4 T cells, which, in turn, differentiate into several subsets of effector cells that have different functions: T helper type I (Th1), Th2, Th17, T follicular helper (Tfh) and regulatory T (Treg) cells. Pro-inflammatory Th17 cells produce IL-17, IL-22 and IL-23, recruit neutrophils and promote inflammation. RAR-related orphan receptor gamma (RORγt) promotes thymocyte differentiation into Th17 cells. By contrast, Treg cells produce anti-inflammatory cytokines IL-10 and TGF-β, suppress activity of a variety of immune cells, inhibiting immune responses (8, 9). Recent studies have identified intestinal RORγt+ Treg cells and found that these microbiota-induced Treg cells were required for the regulation of specific T helper cells in the gut. Recent studies have identified intestinal RORγt+ Treg cells and found that these microbiota-induced Treg cells were required for the regulation of specific T helper cells in the gut (10).

Innate lymphoid cells (ILCs) are recent additions to the innate immune cell family by secreting effector cytokines and regulating the functions of other immune cells (11). Arising from a common lymphoid progenitor and similarly to T cells, they can be further subdivided into 3 main groups: group 1 ILCs that includes the well characterized NK cells, as well as the non-cytotoxic ILC1s, group 2 ILCs or ILC2s, and finally group 3 ILCs including ILC3s and lymphoid tissue inducer cells (12, 13).

Among the vast numbers of commensal bacteria species inhabiting the gastrointestinal tract, a special role is played by certain types of key immunoregulatory bacteria, which direct the differentiation of various T-cell subpopulations; in turn, the TCR of these immune cells appear to be commensal specific (14). In this context, segmented filamentous bacteria (SFB) induce differentiation of type 17 T-helpers (Th17); accordingly, most SFB-induced Th17 cells are specific to SFB antigens. Using hybridoma TCR in SFB-colonized mice, Yang and colleagues showed that most TCR Th17 cells recognize SFB antigens specifically (15).

Among the 19 clusters of Clostridia class identified in the intestines (from 1 to 14), the representatives of clusters IV and XIVa (also known as the groups *Clostridium leptum* and *Clostridium coccoides* respectively) possess the highest Treg-inducing potential. *Clostridium* cluster XIVa encompasses species of the genera *Clostridium, Eubacterium, Ruminococcus, Coprococcus, Dorea, Lachnospira, Roseburia* and *Butyrivibrio*. *Clostridium* cluster IV consists of the genera *Clostridium, Eubacterium, Ruminococcus* and *Anaerofilum* (16). In particular, Treg-inducing activity in mice was detected in a mix of 46 strains of *Clostridium* clusters IV and XIVa (17). A mix of 17 human Clostridia strains with Treg induction activity was also reported (18).

One of the representatives of *Clostridium* cluster IV is the species *Faecalibacterium prausnitzii*, which affects the balance of Th17/Treg in the gut-associated lymphoid tissue (GALT). *F. prausnitzii* is one of the main producers of butyrate in the intestine and can reduce its inflammation by stimulating the large-scale production of IL-10. *F. prausnitzii* also blocks the activation of nuclear factor NF-κB, leading a suppression in the production of pro-inflammatory cytokines IFN-γ, TNF-α, IL-1β, IL-8, IL-12 and increase of the activity of Foxp3^+^ Treg activity in GALT. A protein called the microbial anti-inflammatory molecule (MAM) identified in the supernatants of *F. prausnitzii* culture has been shown to induce IL-10 production *in vitro* and block the development of Dextran sulfate sodium-induced colitis (19).

Representatives of the phylum *Bacteroidetes, Bacteroides fragilis* and *Bacteroides thetaiotaomicron* are also inducers of T regulatory cell differentiation in the gut (20). Important metabolites of *B. fragilis* are short chain fatty acids (SCFAs), which act as a link between the microflora and immune system, activating GALT cells through the FFAR2 receptor. A decrease in the concentration of SCFAs such as butyrate, propionate and acetate reduces the number of Treg in the gut upsetting the Th17/Treg balance, and the level of mRNA FFAR2 changes with the development of experimental inflammatory bowel disease (IBD) (21).

In a previous study, we showed that the introduction of *B. fragilis* to the animals that received vancomycin treatment prior to being infected with *S.* Typhimurium increases the level of mRNA FFAR2 in GALT, affects the expression of *Salmonella* effector proteins SipA, SopB, SopE2 (22), and also leads to an increase in the concentration of SCFAs, helping to reduce *Salmonella*-induced inflammation (23).

The immunoregulatory potential of *Akkermansia muciniphila*, a mucin-degrading bacterium from the phylum *Verrucomicrobia*, is also of a great interest. Plovier and co-authors showed that the introduction of *A. muciniphila* or its external membrane protein Amuc_1100 activates type 2 TLR (TLR2), signalling, which increases the expression of genes encoding tight contact proteins claudin 3 and occludin (24).

In addition to the immunoregulatory potential of these commensal bacteria, another area of focus is their ability to increase the resistance to pathogen colonization and invasion resulting from competition for metabolites, production of inhibitory substances and the development of immune reactions in GALT.

Therefore, the aim of the study was to determine the levels of key immunoregulatory bacteria of the intestinal wall-associated microflora and its effect on the transcriptional activity of the Foxp3 and RORyt genes in GALT of rats with *Salmonella*-induced inflammation, both untreated and treated with vancomycin and *B. fragilis.*

## MATERIALS AND METHODS

The experimental animals, white Wistar mature male rats (n=120) were housed under standard conditions, with proper diet and water *ad libitum* at the animal facility of Zaporizhzhia State Medical University. Animal treatment and all experimental procedures were performed in compliance with the European Convention for the Protection of Vertebrate Animals used for Experimental and other Scientific Purposes. The study was approved by the Ethical Committee of Zaporizhzhia State Medical University.

Experimental study design comprised eight groups: Group 1 – control animals (n=15); group 2 – Vancomycin (animals that were injected orally with vancomycin at a dose of 50 mg/kg) (n=15); group 3 – *S.* Enteritidis (animals that were injected orally with *S.* Enteritidis in the amount of 3 × 108 colony-forming units per 1 ml (CFU/ml) (n=15); group 4 – *S.* Typhimurium (animals that were injected orally with *S*. Typhimurium in the amount of 3 × 10^8^ CFU/ ml) (n=15); group 5 – Vancomycin + *S.* Enteritidis (animals that received vancomycin in a dose of 50 mg/kg and, a day later, received a bacterial load of *S.* Enteritidis in the amount of 3 × 10^8^ CFU/ml) (n=15); group 6 – Vancomycin + *S.* Typhimurium (animals that received vancomycin at 50 mg/kg with per os and a day later received *S.* Typhimurium bacterial load in the amount of 3 × 10^8^ CFU/ml) (n=15); group 7 – Vancomycin + *S.* Enteritidis + *B. fragilis* (animals that received vancomycin at a dose of 50 mg/ kg, a day later - a bacterial load of *S.* Enteritidis in the amount of 3 × 10^8^ CFU/ml and the next day received *B. fragilis* in the amount of 3 × 108 CFU/ml) (n=15); group 8 – Vancomycin + *S.* Typhimurium + *B. fragilis* (animals that received vancomycin at a dose of 50 mg/kg, a day later – a bacterial load of *S*. Typhimurium in the amount of 3 × 10^8^ CFU/ml and the next day received *B. fragilis* in the amount of 3 × 10^8^ CFU/ml) (n=15).

Animal euthanasia was carried out on the fifth day of the experiment by cardiac puncture under deep anaesthesia, in accordance with the requirements of the Animal Care Committee (25).

Animals were infected daily with *Salmonella* cultures grown on 1.5% meat peptone agar obtained from the Museum of Microorganism Strains of the Ukrainian Centre for Disease Control and Monitoring of the Ministry of Health of Ukraine and grown on nutrient media.

Bacterial suspensions were prepared in standard tubes with an external diameter of 18 mm and standardized using a DEN-1B densitometer (Biosan) according to McFarland (McF).

For the administration of *S.* Enteritidis, *S.* Typhimurium and *B. fragilis* to rats, we prepared suspensions at a concentration of 1.0 of McF standard, which corresponds to a concentration of 3 × 10^8^ CFU/ml and injected them 1 ml at a time through an intragastric tube measuring 16–18, length 5–7.5 mm, tip size 2.25.

Molecular genetic studies to determine genus and species of the bacteria, as well as their quantity in total rat microflora, were carried out using quantitative PCR (Q PCR) and 16S rDNA genes as a reference. We used the intestinal wall-associated microflora as a substrate for Q-PCR microbiome studies. Scrapes from the ileum mucosa were lysed in ASL buffer (QIAGEN). Isolation of total DNA from scrapes was carried out according to the manufacturer’s instructions. Intestinal bacterial abundance was measured with Q-PCR on a CFX-96 thermocycler (BioRad) using SYBR Green Master Mix (Thermo Scientific) and specific species and genus-specific primers (Thermo Scientific), (Table 1). Molecular genetic studies to determine genus and species of the bacteria, as well as their quantity in total rat microflora, were carried out using Q PCR and 16S rDNA genes as a reference. We used the intestinal wall-associated microflora as a substrate for Q-PCR microbiome studies. Scrapes from the ileum mucosa were lysed in ASL buffer (QIAGEN). Isolation of total DNA from scrapes was carried out according to the manufacturer’s instructions. Intestinal bacterial abundance was measured with quantitative PCR on a CFX-96 thermocycler (BioRad) using SYBR Green Master Mix (Thermo Scientific) and specific species and genus-specific primers which were selected using PrimerBlast (www.ncbi.nlm.nih.gov/tools/primer-blast) and synthesised by Metabion (Germany) and ThermoScientific (the USA), (Table 1).

**Table 1.** Primers for the determination of bacterial groups due to 16S rDNA

**Bacterial groups**	**Primers**
16S universal	UniF340: 5′-ACTCCTACGGGAGGCAGCAGT-3′
16S universal	UniR514: 5′-ATTACCGCGGCTGCTGGC-3′
*Segmented filamentous bacteria (SFB)*	SFB736F: 5′-GACGCTAGGCATGAGAGCAT-3′
*Segmented filamentous bacteria (SFB)*	SFB844R: 5′-GACGGCACGGATTGTTATTCA-3′
*Akkermansia muciniphila*	F: 5′- CAG CAC GTG AAG GTG GGGAC-3′
*Akkermansia muciniphila*	R: 5′-CCT TGC GGT TGG CTT CAG AT-3′
*Faecalibacterium prausnitzii*	F: 5′-CCC TTC AGT GCC GCA GT-3′
*Faecalibacterium prausnitzii*	R: 5′-GTC GCA GGA TGT CAA GAC-3′
*Bacteroides-Prevotella* group	Bac303F GAAGGTCCCCCACATTG
*Bacteroides-Prevotella* group	Bac708R CAATCGGAGTTCTTCGTG
Clostridial cluster IV	F: TAACACAATAAGTAATCCACCTGG
Clostridial cluster IV	R: ACCTTCCTCCGTTTTGTCAAC
Clostridial cluster XIV	F: CGGTACCTGACTAAGAAGC
Clostridial cluster XIV	R: AGTTTCATTCTTGCGAACG

Transcriptional activity of the Foxp3 and RORγt genes in GALT was determined using real-time reverse transcription polymerase chain reaction. RNA was isolated from the grouped lymphoid nodules (Peyer’s patches) of rat ileum. Total RNA isolation was performed using Trizol RNA Prep 100 (Izogen, Russia). Reverse transcription and cDNA synthesis were performed using Reagent Kit for Reverse Transcription RT-1 (SINTOL, Russia).

To determine expression level of the genes Foxp3 of rats and Rorc (Roγt), we used Maxima SYBR Green qPCR MasterMix (2×) reagent kit (Thermo Scientific, USA) and CFX96™ Real-Time PCR Detection Systems amplifier (Bio-Rad, USA). The final reaction mixture for amplification contained SYBR Green dye, Maxima HotStartTaq DNA Polymerase, 0.2 μl of direct and reverse specific primers, and 1 μl of cDNA template. The reaction mixture was brought up to a total volume of 25 μl with dionized H_2_O. Specific primer pairs (5′-3′) to the studied and reference genes were selected using PrimerBlast and synthesised by Metabion (Germany) and ThermoScientific (the USA), (Table 2).

**Table 2.** Primers for determining the level of genes expression of Foxp3 and Rorc (Royt) in rats

**Gen symbol**	**Primers**
Foxp3	F = CGAGACTTGGAAGTCAGCCAC; R = TCTGAGGCAGGCTGGATAACG
Rorc (Royt)	F = AACATCTCGGGAGTTGCTGG; R = TCGATTTGTGAGGTGTGGGT
GAPDH	F = GCCTGGAGAAACCTGCCAAG; R = GCCTGCTTCACCACCTTCT

Quantitative results were normalized to universal 16S rDNA and analysed using the ΔCt method. Statistical analysis was performed on GraphPad Prism (GraphPad Software) and StatSoft Statistica v12 (NAXXR712D833214FAN5) using the nonparametric Mann - Whitney test (U-test) to calculate significance of differences between the means of the results were considered significant at *p*≤0.05. The relative normalized amount of cDNA target genes was determined by the comparative method ΔΔCt. Statistical analysis of PCR data was performed using CFX Manager™ software (Bio-Rad, USA). The experiment included the following negative controls: no template cDNA in the PCR reaction, on template mRNA in the synthesis of cDNA, and no fragments in the synthesis of cDNA. All amplification reactions were performed on individual samples in triplicate.

## RESULTS

When experimental animals received vancomycin (group 2), the numbers of SFB species significantly increased vs control group (*p*≤0.0001). The number of A. muciniphila (*p*≤0.0001), *F. prausnitzii* (*p*≤0.0017), *Bacteroides* + *Prevotella* (*p*≤0.0005) representatives significantly decreased while the decrease in *Cl*. cluster IV species (*p*≤0.0046), was less pronounced compared to the control.

In rats infected with *S.* Enteritidis and *S.* Typhimurium (groups 3 and 4), the number of *SFB* (*p*≤0.0001) species increased and the number of *A. muciniphila* (*p*≤0.0001; *p*≤0.0001) and *Bacteroides* + *Prevotella* (*p*≤0.0002; *p*≤0.0002) species decreased *vs*. the control group.

When the animals were treated both with vancomycin and *Salmonella* we observed more pronounced changes in the species composition of these genera of intestinal wall-associated bacteria. Accordingly, in the experimental groups 5 and 6 we found a sharp decrease in the number of *A. muciniphila* (*p*≤0.286; *p*≤0.0055), *F. prausnitzii* (*p*≤0.0011 and *p*≤0.0002, respectively). The numbers of *SFB* (*p*≤0.0001; *p*≤0.0001), *Bacteroides* + *Prevotella* (*p*≤0.0189; *p*≤0.0017), *Cl*. ñluster IV (*p*≤0.0024; *p*≤0.0033), *Cl.* ñluster XIV (*p*≤0.0277 and *p*≤0.0198, respectively) species significantly increased.

Combined administration of vancomycin, *Salmonella* and *B. fragilis* (group 7 and 8), caused a pronounced increase in *Bacteroides* + *Prevotella* (*p*≤0.0001; *p*≤0.0001), *Cl*. ñluster IV (*p*≤0.0019; *p*≤0.0005) and *Cl*. ñluster XIV (*p*≤0.0151 and *p*≤0.0021, respectively) species compared to the groups 5 and 6. The number of *A. muciniphila* and *F. prausnitzii* species significantly increased only in the group 8 (*p*≤0.0166; *p*≤0.0001) while the number of *SFB* species significantly decreased (*p*≤0.0001 and *đ*≤0.0001, respectively) compared to the groups 5 and 6 (Table 3).

**Table 3.** The average content of intestinal wall-associated microflora in rats identified by 16 rDNA

**Groups**	***Akkermansia muciniphila* phylum Verrucomicrobia**			***Faecalibacterium prausnitzii* phylum Firmicutes**			***Akkermansia muciniphila* phylum Verrucomicrobia**		
		
	16S (CT mean)	SFB (CT mean)	2^-ΔCT	16S (CT mean)	F. prausn. (CT mean)	2^-ΔCT	16S (CT mean)	Akk. muciniph. (CT mean)	2^-ΔCT
Group 1	10.06	27.91	0.000005	9.74	22.93	0.00015	10.17	21.79	0.00051
Group 2	10.03	22.02	0.00025^*^	10.09	28.98	0.0000028^*^	10.17	27.76	0.0000083^*^
Group 3	9.94	21.0	0.00047^*^	10.32	23.25	0.00018	10.22	26.57	0.000017^*^
Group 4	9.92	20.13	0.00089^*^	9.96	22.98	0.00015	10.05	26.24	0.000018^*^
Group 5	10.12	17.45	0.0067^a^	10.28	30.24	0.0000014^a^	9.95	28.81	0.0000025^a^
Group 6	9.91	17.09	0.0075^b^	10.24	30.04	0.0000015^b^	10.20	28.97	0.0000029^b^
Group 7	10.01	30.12	0.00000093^c^	10.05	18.26	0.0068	9.73	17.99	0.0046
Group 8	10.02	31.05	0.00000051^d^	9.98	18.20	0.0054^d^	9.90	18.01	0.0039^d^

**Groups**	***Clostridium* cluster IV**			***Clostridium* cluster XIV**			***Bacteriodetes: Prevotella* cluster**		
		
	16S (CT mean)	*Cl.* Cluster IV (CT mean)	2^-ΔCT	16S (CT mean)	Cl. ÑlusterX IV (CT mean)	2^-ΔCT	16S (CT mean)	Bact.-prev. gr (CT mean)	2^-ΔCT
Group 1	9.96	15.54	0.029	10.02	15.28	0.045	9.83	14.17	0.091
Group 2	10.07	17.81	0.0064^*^	10.10	16.72	0.014	9.71	17.11	0.0077^*^
Group 3	10.13	16.44	0.017	9.82	16.33	0.021	9.94	18.93	0.0032^*^
Group 4	10.25	16.03	0.029	9.89	16.10	0.024	10.41	19.17	0.0032^*^
Group 5	10.07	19.99	0.0014^a^	9.88	21.04	0.00065^a^	10.07	22.27	0.00026^a^
Group 6	10.27	20.07	0.0014^b^	10.10	20.99	0.00066^b^	9.96	22.18	0.00029^b^
Group 7	10.01	12.65	0.22^c^	9.53	13.89	0.076^c^	9.88	12.56	0.187^c^
Group 8	9.65	12.75	0.15^d^	9.33	13.86	0.062^d^	10.17	12.64	0.212^d^

* - the reliability of the differences in the parameters *p*<0.05 *vs* control group;

a - the reliability of the differences in the parameters *p*<0.05 *vs* group *S.* Enteritidis;

b - the reliability of the differences between the parameters *p*<0.05 *vs* group *S*. Typhimurium;

c - the reliability of the differences between the parameters *p*<0.05 *vs* group *S.* Enteritidis + Vancomicin;

d - the reliability of the differences in the parameters *p*<0.05 in *vs* group *S*. Typhimurium + Vancomicin.

**Fig. 1.**
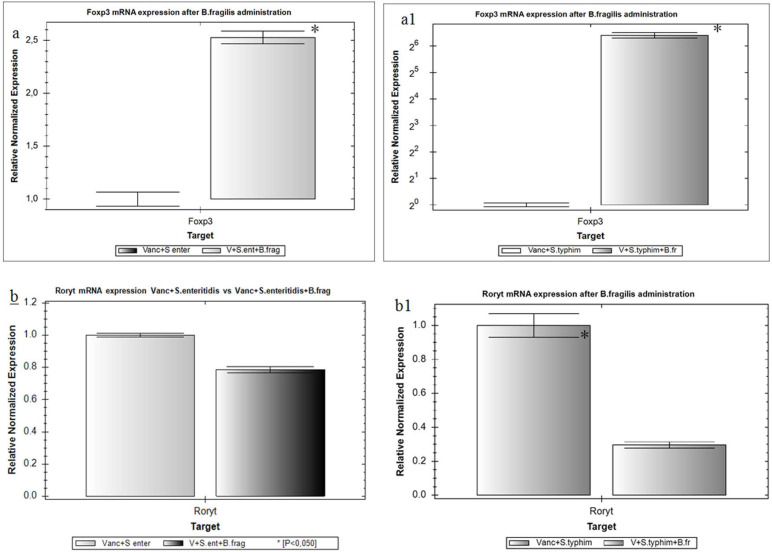
Relative amount of mRNA Foxp3 (a, a1) and RORγt (b, b1) genes in grouped lymphoid nodules of the ileum of rats when *B. fragilis* was administered to experimental animals treated with *S*. enteritidis and *S*. Typhimurium on the background of pre-treatment with vancomycin. Normalization by the ΔΔCt method with the GAPDH reference gene. ^*^ - *p*<0.05

Since *B. fragilis* is one of the main producers of SCFAs which activate GALT cells, we determined the level of mRNA of the Foxp3 transcription factor, directs protein directing T cell differentiation into Treg. Transcriptional activity of the Foxp3 gene in the groups 3, 4 and 6 remained unchanged, while it significantly decreased in the group 5 (by 67%, *p*≤0.05). However, with the administration of *B. fragilis*, the relative amount of Foxp3 mRNA in the groups Vancomycin + *S*. Enteritidis + *B. fragilis* and Vancomycin + *S*. Typhimurium + *B. fragilis* increased 2.5 and 85 times, respectively (*p*≤0.05) (Fig. 1a, a1). An increase in the expression levels of the transcription factor RORγt, which regulates differentiation of Th17 subpopulation, was fo und in the groups 3, 4, 5 and 6 (2.9, 3.9, 13.0 and 2.5 times respectively) while it significantly decreased in the group 8 (by 70%, *p*≤0.05), (Fig. 1b, b1).

**Fig. 2.**
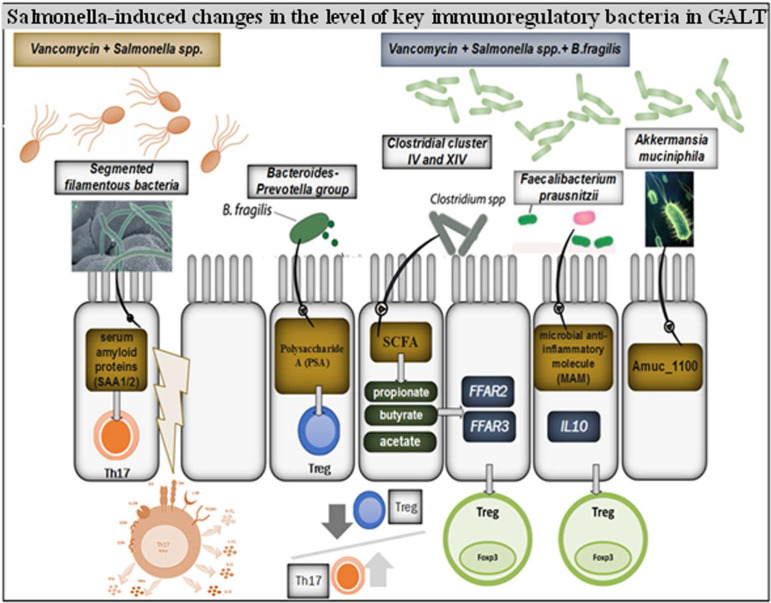
*Salmonella*-induced changes in the levels of key immunoregulatory bacteria in GALT

Our results indicate the possibility of using *B. fragilis* to correct salmonella-induced changes in the intestinal microbiome. We observed a decrease in *SFB* level and increase in *F. prausnitzii, A. muciniphila, Cl.* ñluster IV, *Cl.* ñluster XIV and *Bacteroides* + *Prevotella* levels (Fig. 2).

## DISCUSSION

Some species of the intestinal microflora such as: Segmented Filamentous Bacteria, *Faecalibacterium prausnitzii, Akkermansia muciniphila, Clostridium* cluster IV, *Clostridium* cluster XIVa, *Bacteroides fragilis*, regulate activation of congenital and adaptive units of immune response in GALT, which, in turn, can affect outcome of the infection (26).

There is a complex and dynamic interplay between the intestinal microbiota and differentiation of CD4 + T cells, shaping adaptive immune responses in normal conditions and inflammation (27, 28).

Production of Th17 cells in the small intestine can increase in response to colonization by *SFB* or the altered Schaedler flora (ASF); the extent of this production often correlates with the quantities of Clostridia and other commensals. The question of how *SFB* primarily induce Th17 differentiation was addressed in two recent studies, which have shown that a key feature of *SFB* required for Th17 induction is close association of the bacteria with the intestinal epithelial cells. Using strains of *SFB* specific to mice and rats, Atarashi and colleagues found that only strains obtained from the original host were able to induce Th17 cells, and possessed connected species-specific adhesion mechanisms (17). *SFB* adhesion in epithelial cells induced expression of epithelial serum amyloid proteins (SAA1/2) and reactive protein producing oxygen Dual oxidase 2 (Duox2). SAA1 promotes the induction of IL-17A and IL-17F by Th17 cells; it also acts on dendritic cells, promoting Th17 induction and increasing RORγt+ expression. Additionally, IL22, canonical Th17 cytokine, induces the production of antimicrobial peptides that are effective against bacteria such as *C. rodentium* and some strains of *E. coli, S.* Typhimurium, and *Yersinia enterocolitica* (29).

Group 3 Innate Lymphoid Cells (ILC3s) are key sentinels of barrier tissue homeostasis and critical regulators of host-commensal mutualism—and respond rapidly to damage, inflammation and infection to restore tissue health (30). ILC3s (like Th17) are characterized by the expression of the retinoid-related orphan receptor γt (RORγt). Therefore, part of RORγt-expressing cells, certainly could be ILC3, but not Th17. The methodology used by us does not allow us to distinguish Th17 from ILC3. In line with these findings, ILC3s have the capacity to crosstalk both directly and indirectly with the adaptive immune system through the production of multiple soluble factors. Following exposure to the commensal microbiota IL-22 produced by ILC3s acts to support homeostatic tissue Th17 responses through the induction of serum amyloid protein A (SAA) from epithelial cells) (29). ILC3s participate in the immune responses to *Citrobacter rodentium, Clostridium difficile, Salmonella enterica, Listeria monocytogenes* and *Toxoplasma gondii* (11). Qiu J et al. has shown that ILC3s restrict colonization of segmented filamentous bacteria in the gut (31) and inhibit the invasion of *Alcaligenes* species into Peyer’s patches (32). ILC3s counter bacterial infections through secretion of IL-22, which stimulates epithelial cells to produce antimicrobial peptides (33).

Colonization by SFB was found to increase expression of major histocompatibility complex (MHC) class II molecules on intestinal epithelial cells and affect the glycosylation of enterocytes, promoting expression of GM1 (monosialotetrahexosylganglioside) glycolipids responsible for inhibiting the attachment of another agent (34).

Our results correspond with other studies of *Bacteroides fragilis* colonization of the intestine involving its polysaccharide A (PSA), which demonstrated that it induces Tregs and IL-10 production via TLR2 signalling (35). In addition, bacterial metabolites, such as short chain fatty acids (SCFAs), can also regulate production of Treg cells and increase acetylation of the Foxp3 locus in Tregs (36). A study of mice colonized with 17 human *Clostridium* strains, found increased levels of SCFAs (acetate, propionate, isobutyrate and butyrate), stimulating production of the suppressor cytokine TGFβ in the epithelial cells of large intestine and indirectly contributing to the development of Tregs. Receptors activated by SCFA, namely G-protein (GPCR) associated GPR43 (FFAR2), are expressed both in the epithelial cells and in most hematopoietic cells. Accordingly, mice lacking GPR43 had lower levels of Tregs (37).

Lee Y. K. et al. found that gut inflammation caused by dextran sulfate sodium treatment was inhibited by *B. fragilis* colonization in mice. Further, they reveal a protective role of *B. fragilis* treatment against colon tumorigenesis using an azoxymethane/dextran sulfate sodium-induced model of colitis-associated colon cancer in mice (38). Researches suggest that protective role against tumor formation provided by *B. fragilis* is associated with inhibition of expression of the chemokine receptor C-C chemokine receptor 5 (CCR5) in the colon. The molecular mechanism for protection against colorectal cancer (CRC) provided by *B. fragilis* is dependent on polysaccharide A (PSA) production and is mediated by TLR2 signaling. When PSA in *B. fragilis* is destroyed, they lose their ability to induce Tregs and associate with intestinal epithelium, suggesting the importance of Tregs for the colonization of *B. fragilis* in this niche. The PSA action is an example of a powerful therapeutic potential that can be revealed by comprehensive study of commensal microorganisms (39).

At the same time, there are recent data indicated that enterotoxigenic *B. fragilis* strains (ETBF) producing *B. fragilis* toxin (BFT) are associated with CRC, especially in the patients with precancerous lesions and initial carcinogenic lesions (40) and with ulcerative colitis, especially in the patients with diarrhea symptoms (41). Furthermore, some studies have been published recently, describing ETBF as a trigger for chronic Stat3/IL-17-driven colitis which is IL-17 dependent and induces T17 colitis and tumorogenesis through the secretion of the metalloproteinase Bft and other associated factors (42).

In general, commensals can regulate immune specificity through a number of mechanisms: a) microorganisms in the intestine can induce populations of T cells to express two TCRs: one TCR recognizes the commensal itself, and the other responds to its own peptide. The migration of these T cells into the host tissue can produce pathological and autoimmune responses; b) CD4 + and CD8 + T cells can be activated independently of their TCR via Toll-like receptors (TLRs). In particular, stimulation of TLR on T cells by bacteroids facilitates their differentiation into Treg; c) commensal microorganisms can express epitopes that mimic their own peptides, which are known to drive autoimmunity. Own reactive T cells will be activated by these antigens in the intestine, and then enter the tissues causing autoimmunity (43).

Commensal intestinal microbiota provides resistance to colonization by pathogenic microorganisms. Mice infected with *S. typhimurium*, show higher resistance to pathogens when pre-treated with an antibiotic, because in this case there is less disruption to the resident microbial community. If such disruption does occur, it can lead to an intensified invasion by salmonella and in humans’ development of chronic asymptomatic carrier status. Commensal *Bacteroides* spp. can to limit salmonella-induced infection of the intestine due to the production of SCFA such as propionate, acetate and butyrate. However, salmonella may avoid innate and adaptive immunity response by producing effector proteins that facilitate introduction, survival and auto-reproduction of bacteria in the body tissues, which leads to intestinal inflammation (44–46).

Studies demonstrate that introduction of live *A. muciniphila* increased endogenous production of specific biologically active lipids, had anti-inflammatory effect and regulated endogenous production of intestinal peptides, glucagon-like peptides 1 and 2 (GLP-1 and GLP-2), involved in the regulation of glucose and intestinal barrier function (47). High level of metabolites produced by Clostridia species can contribute to the development of Peyer’s plaques, αβ-TCR intraepithelial lymphocytes (IEL) and the production of IL-7, IL-6, TGFβ, IgA, in addition to affecting the ratio of CD4-CD8 + and CD4 + CD8-lymphocytes (17). Furthermore, *Clostridium* spp. XIV and IV clusters induced the accumulation of Tregs cells in their stratum of the intestinal mucosa by activating expression of Foxp3 transcription factor and IL-10. Matrix metalloproteinases (MMP) produced by these species, activated TGF-β and indolamine 2,3-dioxigenase (IDO) in the epithelial cells of large intestine and reduced the number of Th17. These actions, helped to maintain natural and adaptive immune homeostasis in the intestine (48, 49).

Currently, only some commensal microbe species are recognised as being capable of modulating specific immune parameters, but recent studies suggest that a wide range phylogenetically diverse human intestinal microbes possess such immunomodulating effect. Most bacteria exert several specialized, additive, or excessive transcriptional and immunomodulatory effects. Interestingly, there is little correlation with the phylogenetic position of the microbes. Additional studies have a potential to identify new important therapeutic effects.

Screening of the intestinal microbiome has already has produced a number of discoveries, both expected and unanticipated. For example, some microorganisms were found to be capable of inducing Th17 cells in the intestinal epithelium to an extent similar to that of SFB In a rather surprising finding, about a quarter of the studied bacteria, and representing diverse phyla, can induce RORyt + Helios-Tregs in the large intestine (50). Another previously unknown, but potentially interesting immunomodulatory activity of *Veillonella* species is stimulating an increase in IL 10-producing CD4 + T cells and parallel decrease in IL 22-producing epithelial cells in the large intestine. *L. Rhamnosus* significantly reduces the number of dendritic cells, while *F. Varium* produces an unusually strong immune-stimulating activity (51).

## CONCLUSION

Separate administration of vancomycin and salmonella to animals caused quantitative changes in the levels of key immunoregulatory bacteria: an increase in SFB species and a significant decrease of *A. muciniphila* and *F. prausnitzii* species. Rats previously treated with vancomycin and subsequently infected with *S.* Enteritidis and *S*. *typhimurium*, more pronounced changes in the quantitative composition of the microbiota were observed, leading to the decrease Foxp3+ and increase in Rorγt+ mRNA levels. However, the introduction of *B. fragilis* to the experimental animals inoculated with *S*. Enteritidis or *S.* Typhimurium against the background of pre-treatment with vancomycin, reduced the levels of *SFB* and Rorγt+ mRNA, and significantly increased the proportion of the *Bacteroides-Prevotela* group, *A. muciniphila, Clostridium* spp. clusters XIV, IV, *F. prausnitzii* as well as Foxp3+ gene expression, indicating restoration of intestinal microbiome homeostasis. Our results suggest that the commensal microorganism *B. fragilis* may provide a protective role against the development of experimental colitis, which has to be taken into consideration for further clarification of the effective therapeutic strategy of inflammatory bowel diseases, irritable bowel syndrome and necrotising colitis.
